# How Authenticity and Tradition Shift into Sustainability and Innovation: Evidence from Italian Agritourism

**DOI:** 10.3390/ijerph17155389

**Published:** 2020-07-27

**Authors:** Pamela Palmi, Greta Enrica Lezzi

**Affiliations:** 1 Department of Economics and Management, University of Salento, Ecotekne, Prov.le Lecce-Monteroni, 73100 Lecce, Italy; pamela.palmi@unisalento.it; 2 Department of Lecce, Regional Agency for the Prevention and Protection of the Environment, Via Miglietta 2, 73100 Lecce, Italy

**Keywords:** innovation, tradition, source of tradition, authenticity, sustainability, agritourism

## Abstract

This study investigates the topic of innovation strategies based on tradition in the wake of sustainability, in the agritourism sector, as derived from the phenomenon of multifunctionality in agriculture. The results reveal that the tangible and intangible resources that originate from tradition are drivers for innovation. The research highlights how tradition, as grounded on diverse foundations, is able to generate novel products and services stemming from an innovative arrangement of past events, particularly the identity of a place, which brings out its authenticity and makes it even more attractive. In this paper, we delve into the multifaceted outcomes that tangible and intangible traditions have on the innovation and distinctive standing of this rising accommodation offer with regard to post-productivism agriculture, and how this is accomplished while also looking for sustainability. The sourced dataset is based on a qualitative investigation of 10 cases in the Salento area of Puglia, a region of southern Italy. Tradition-grounded strategies proved to have several viable routes leading to innovation and bear positive impacts on the territory and the creation of value, yielding significant results both for scholars and practitioners.

## 1. Introduction

In recent years, the agritourism sector has been a successful case of the Made in Italy tourism offer, perhaps representing the most important and radical product innovation that has affected Italian agriculture, with renewed and winning business models [1].

Today, around 23,000 farms offer agritourism services, for a yearly turnover that now exceeds one billion euros [2]. Agritourism and rural tourism are phenomena affecting most of the advanced development countries [3,4,5,6] and intercept the global evolution of tourism demand [7]. However, nowhere in the world have they assumed the dimensions, the refinement, and the quality level that we register today in Italy [1,8,9,10,11], also thanks to the top–down impulse, Italy being the only country in Europe that has a specific law on agritourism [12].

Therefore, agritourism in Italy is now everything, except a new and niche phenomenon. For this reason, it is useful to put oneself in a more analytical and critical way towards thinking about it, trying to understand what changes are underway, what happened already, and even more so, what may be in the near future—which the key factors will generate further development and new value, especially in the wake of sustainability.

In recent years, the multifunctional vision of agriculture has greatly helped the development of this sector [13,14,15,16]. The conceptualization of post-productivism agriculture, no longer seen only as the production of primary goods but also as the ability to provide services with a series of positive externalities that impact on the territory they belonged to, allowed to combine primary agriculture with the new needs of the community [17]. A wide range of services has been added to the production of food and fiber, seeking solutions that create value, combining strictly productive objectives with social and environmental ones [18].

Both scholars and policymakers have recognized the importance of multifunctionality in agriculture, “sustaining rural landscapes, protecting biodiversity, generating employment and contributing to the viability of rural areas” [19]. In fact, the concept of agricultural multifunctionality [15,20] focuses on rural development and agro-ecology, underlining the role of agriculture in supporting rural economies and local communities. In this perspective, farmers are strongly connected with other local actors through the social and environmental functions of their business [21].

Agritourism is increasingly absorbing this conceptualization, and well reflect the changes in society on a global scale; it captures the key factors for value creation within the three sustainability dimensions of the multifunctional perspective: economic, sociocultural, and environmental [4].

In this framework of renewal stimuli and evolutionary possibilities, several studies underline the economic potential of “agritourism” as a farm diversification strategy [3,4,5,6,22,23,24,25,26,27,28,29,30].

Moreover, the benefits of agritourism are not limited to the private economic gain for entrepreneurs, but also generate social benefits, such as helping health and wellbeing [31], the sustainable development of rural areas [32], as well as the recovery of authenticity and tradition that generate public value.

Therefore, two interrelated issues emerge to which policymakers should pay attention. The first concerns a new vision of the territory as a driver for social relations and interdependencies that can promote more or less radical social changes oriented towards sustainability, not only in business models but also in ways of consuming and living [33,34,35,36]. The second concerns the spatial dimension of sustainability that shifts the focus on local development processes, strengthening the link between business and territory [37,38].

It is quite evident that, in recent years, the phenomenon of agritourism has been increasingly developing strong interdependencies between the agricultural and tourism sectors, particularly with experiential tourism [39,40]. This phenomenon can be considered an expression of a far-sighted entrepreneurship, which, if encouraged, can fulfill a great role from the perspective of the wanted sustainable development [41].

Recent studies have underlined the importance of innovation to guarantee a sustainable competitive advantage in any sector. However, innovation becomes especially crucial in sectors where markets are overloaded by a wide variety of offers, pushing customers to resort to products or services from worldwide providers, as is the case for the holiday sector in general and, therefore, also for agritourism [42,43,44,45].

Agritourism is challenged by major issues, as a consequence of the evolving demographic trends of travelers’ lifestyles and somewhat easier methods of replicating the accommodation offer [46,47,48,49,50]. In this context of widespread challenges, in order to improve the competitive advantage of our tourism enterprises, and specifically agritourism, new strategies to innovate are to be found [51,52,53] in a global, increasingly competitive and demanding context [54,55,56].

As Brooker and Joppe [57] argue, innovation involves “introducing a new concept, whether it is a product, process, service, marketing technique, organizational structure, or market”. Related to agritourism, innovation is also brought about by products (for instance, not only bio products such as olive oil or wine, but also of medicinal herbs and phytotherapy products; new rural services or new experiences to live on site; and creation of products in synergy with other local players), providers (e.g., new international niche tour operators), new markets (e.g., directing existing destination brands to attract new markets), and new ways to organize a business (e.g., typical cooking courses; local craft courses; local dance courses; and improvement of tours using new technologies that enhance the experience) [47].

Leveraging on past traditions as a foundation towards innovative changes has been widely debated in the international management literature [45,58,59,60,61,62,63]. As a matter of fact, returning to origins and authenticity reveal values, practices, and skills connected to specific and local traditions that can contribute to the development and support of unique or distinct products or services, acquiring a strong identity trait.

Although this vision of the innovation process, defined the “recombinant perspective” [64], has been widely applied across different sectors, also recently in tourism and hospitality [45], the literature shows a gap in the agritourism topic. Given the growing importance that agritourism hosts attach to “authenticity” [65], and more precisely to the locally rooted culture and its very legacy of natural and untouched awareness [66], it is important to analyze the different approaches that organizations implement to innovate the market of alternative tourism.

Within this theoretical context, the purpose of this research is to examine a sample of agritourism to be used as a case study to analyze the connection between tradition and innovation. More in detail, relevance is given to agritourism established in an environment where historical folklore becomes prominent, especially those committed to pursuing the goal of sustainability.

Agritourism, pushed by multifunctionality in agriculture, is above all also a novel way of hospitality, which is highly developed in Italy and rapidly gaining momentum worldwide; indeed, the re-discovery of cultural heritage stemming from ancient peasant civilizations bears and preserves a strong identity [67].

Agritourism is also a formidable tool to leverage on local resources, relaunching artisan arts and crafts, promoting locally sourced products, and fostering interaction between guests and hosts. Therefore, it has recently caught the attention of numerous scholars on the subject [53,68,69,70].

This effort is especially important in view of the general tourism sector, given the increasing need for authenticity when it comes to tourism: consumers aim at attaining original and uncontaminated experiences [71]. Consequently, this research addresses the following questions:

▪ How can agritourism organizations innovate through tradition, in the wake of sustainability?
▪ What foundations can tradition leverage on in the quest for innovative changes in the wake of sustainability?
▪ How does agritourism contribute to the local development process and to create public value?

This research delves into the particular agritourism features and their connections with tradition as a way to push innovation. With these premises, our research makes use of an inductive–deductive methodology, beginning with a description of the relevant framework based on the selected theoretical context. The employed analysis is of a qualitative empirical type, carried out on performed on-field information assembled by observing a group of Italian agritourism establishments in the territory of Salento, in the Puglia Region.

## 2. Agritourism: Evolution of the Phenomenon

### 2.1. Scenario

Currently, agricultural companies represent 95% of the agri-food sector in Italy, the second largest production sector, after that of construction, by volume of revenue. According to multifunctional logic, the diversification activities of income sources represent an increasing share of the Italian agricultural production [72].

The continuous growth of support and secondary activities show that these are not affected by the uncertainty that instead characterizes agricultural production, which is more exposed to factors and exogenous variables (e.g., market instability and price volatility or the succession of unfavorable weather events). Moreover, generally the activities of agritourism are completely in line with the principles of sustainability, which has now become the central element of all development strategies at the EU level (Strategy 2030, New Green Deal, etc.).

In this regard, it is necessary to highlight the stimulus action of the European Union in the promotion of agricultural entrepreneurship and rural development (EU Regulation “Support for rural development”, 2013). The recent Common Agricultural Policy reform also highlights the interest in the revitalization of rural areas, which is considered essential for its fundamental role in the conservation and efficient management of the territory. In this sense, the incentives coming from the Rural Development Programs 2014–2020 are also oriented.

Agriculture is therefore at the center of important changes that are transforming the socio-economic fabric of rural areas, stimulating farms to expand their offerings with services aimed at improving the wider rural nature of the territories in which they are rooted. One of the factors of this change is agritourism, which engages in a new entrepreneurial “mentality” on traditional forms of economic management, sensitive to the demand for services and attentive to the protection of the environment and the landscape, as well as the recovery of the existing building heritage with conservative renovations.

**Figure 1:**
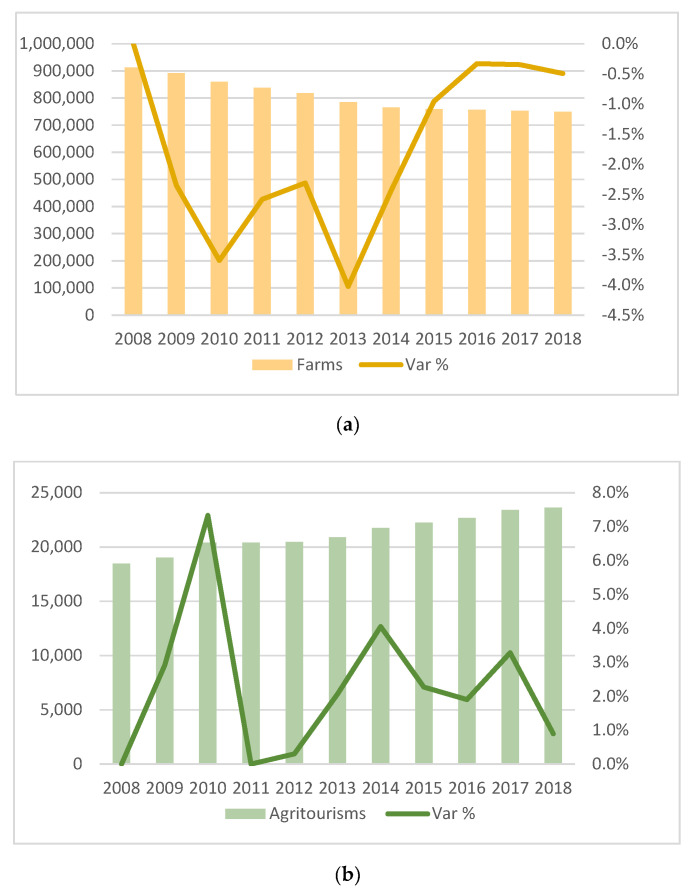
Trends in agricultural enterprises from 2008 to 2018 in Italy: (**a**) farms; (**b**) agritourism. Elaboration based on InfoCamere 2019 [73] and ISTAT 2019 [2].

In the last decade, the evolutionary trends of agricultural enterprises highlight two aspects: a gradual attention to the number of companies in the agricultural sector (−18% from 2008 to 2018) [73] (Figure 1a); and a strong entrepreneurial dynamism in the hospitality sector (+28% from 2008 to 2018) through agritourism [2] (Figure 1b).

The growth trend recorded in recent years concerns both the number of structures and the presence of customers and its economic value. Between 2007 and 2018, the growth of agritourism was over 33%, with an active balance of 5895 companies. In 2018, 23,615 agritourism entities were registered, 0.9% more than the previous year. The number of overnight stays in the agritourism sector went from 8.2 million in 2007 to around 13.4 million in 2018 (+5.2 million), as emerged from the ISTAT survey (2019) [2] on the movement of customers in hospitality establishments.

Between 2007 and 2018, the current value of agritourism production rose from 1.08 to 1.39 billion euros (+29%). Despite the decreases recorded in 2009 and 2012, due to a strong financial and economic crisis, starting from 2013 the production trend is positive. Among the sharply increasing activities, the companies offering tasting initiatives are highlighted (between 2007 and 2018, they grew by 61.3%), as are those offering catering (+36.8%) and accommodation (+30.6%), followed by “other activities”, which showed an increase of 32.5%. The latter class, of particular interest with regard to the evolutionary dynamics of the phenomenon, refers to the expansion by agricultural entrepreneurs of the agritourism offer according to a multifunctional logic. By now, about 55% of the companies are authorized to carry out other agritourism activities, such as excursions, horseback riding, trekking, naturalistic observations, typical local cuisine classes, yoga courses, sports, educational farms, etc. [2].

With regard to the distribution in Italy, agritourism is mainly concentrated in the north (45.1% of the structures), followed by the center (35.5%), the south (12.9%), and the islands (6.5%). Moreover, in the south the most dynamic region is Puglia (+16.5%), in the center there are Umbria and Lazio (+2%), while in the north-east the highest growth is recorded in Veneto (+2.2%) [2].

### 2.2. Tradition as a Driver to Implement Innovation Strategies in the Wake of Sustainability

Examined under a sociological perspective, traditions (from the Latin verb “*tradere*”: to transport, to deliver; something transmitted, handed down) are the aspects of our culture that have been handed down to us from the past, and that we transmit to future generations. “Constellations of symbols”, rites and beliefs but also of particular habits; a corpus of myths and legends; a way of life characteristic of a certain society [74]. Therefore, we can say that traditions are a mix of elements through which it is possible to evoke collective memories, identity, and social cohesion [75].

As Hibbert and Huxman (2010) [76] argue about the organizational theory of the role of tradition, the concept is connected to the stock of knowledge, skills, materials, production processes, signs, values, and beliefs related to the past. Tradition implies the accumulation of know-how, of cultural and symbolic contents, of micro-institutions of practice handed down by innovations that help shape the distinctiveness of individual, organizational, and territorial identity [77,78].

Since tradition is a highly peculiar trait and is therefore difficult to be replicated by other competitors [79], it follows that companies that use traditional approaches can push innovation geared towards distinctiveness. This represents an important wellspring of resources that lead to new earnings [80,81]. Therefore, the process of recombination of tangible resources (such as an ancient building or an art object) or previously existing intangible ones (such as a recipe from the local tradition or an authentic folk dance) [82] can represent the formula for a successful innovation [83].

Researchers have recommended different measurements along which organizations can look for and recombine different assets, namely industrial, organizational, and geographic resources [84,85]. As Petruzzelli and Savino (2015) [62] argue, new products or new services can derive from a competitive mix of local and cultural traditions, leveraging on the legacy of some long-time established small family firms and the implementation of front-line know-hows. This is very powerful, because companies have the opportunity to capture the value related to innovation, generating and supporting the competitive advantage [86].

Despite the relevance of the topic, research has only recently been conducted in the tourism sector to understand the relationship between innovation and strategies based on tradition [45]. However, a survey of the literature on the agritourism highlights a lack of studies in the aforementioned perspective. Therefore, in order to help fill this gap, the present research aims to explore the relationship between innovation, in the wake of sustainability, and strategies based on tradition and authenticity. 

The innovation generated is not unrelated to sustainability, rather it supports and nourishes it. In fact, agritourism is a business model oriented towards sustainability, in its economic, environmental, and social dimensions [1,3,14,22], and it is able to contribute to the sustainable development of rural areas, representing a novel hospitality strategy grounded in sustainable development values [68].

As has been widely demonstrated, agritourism contributes to the achievement of the economic sustainability of the agricultural enterprise, as well as of other economic activities of the territory [4,12,29,30,86,87,88,89,90,91,92,93,94,95,96].

We should take into consideration that environmental sustainability and agricultural businesses, through agritourism, contribute to the conservation and protection of the natural habitat and of the ecosystem; to the rediscovery of ancient types of land products, such as ancient cultivars, traditional medicinal herbs, and ancient vines; to the careful use of resources; and to the revitalization of the countryside through the requalification of the existing real estate structures and the investment in new infrastructures respecting nature and ecology [3,4,10,89,97,98,99,100]. Finally, with regard to the dimension of social sustainability, agritourism contributes not only through the preservation of rural culture and lifestyles, but also through a greater awareness of the value of agricultural and natural heritage—the enhancement of the traditions that are revived through typical quality products that are authentic and healthy [4,11,30,89,92,95,96,101,102,103,104,105,106]. In these kind of structures, tourists live an increasingly genuine and authentic experience, so as to bond with the surrounding community, an experience that turns guests into temporary residents, rather than occasional travelers [107].

For what has been explained so far, the aim of this research is to highlight how, over time, tracking down the significant features of tradition is a way for agritourism to implement innovation in the wake of sustainability. This study is concretely aimed at exploring how agritourism firms can strategically benefit from a shared know-how, as well as from inherited skills, methods, ethics, and principles that come from diverse sources, both inside and outside the farm (as is the case of best practices where shared knowledge and other formulas of inter-organizational cooperation lead to profitable outcomes).

## 3. Research Methodology 

### 3.1. Methodology

Consistent with the aims of the research and with the proposed interpretative framework, the research methodology chosen is that of a case study analysis, according to a qualitative perspective and multiple cases [108,109]. Using multiple cases allows to get more solid results than using a single case [110].

Additionally, and where applicable, multifaceted data collection procedures are here deployed in order to make use of triangulation methods [111]. This methodology has been remarkably beneficial because we could carry on a comparative study across different agritourism establishments—each suitable to be observed as a stand-alone case. A wider examination approach has been necessary due to the relatively new topic under scrutiny, and the perspective of a greater construction of the theory [112].

Moreover, building on Berends and Deken’s formats for the composition of qualitative process research (2019), these cases consist of an empirical narrative that provides the theoretical significance of events and facts that are narrated. Our narrative therefore follows the “conceptual composition” format [113].

### 3.2. Data Gathering 

Research has been carried on through two main phases that followed from October 2018 to November 2019: Phase (1) from October 2018 to February 2019—identification of the number of agricultural enterprises with agritourism activities in the Italian territory and identification of the cases under study, within the Salento area in Puglia, a region of Southern Italy, through the appropriate selection criteria; and Phase (2) from March 2019 to November 2019—collection, analysis, and interpretation of the data and information. 

**Figure 2:**
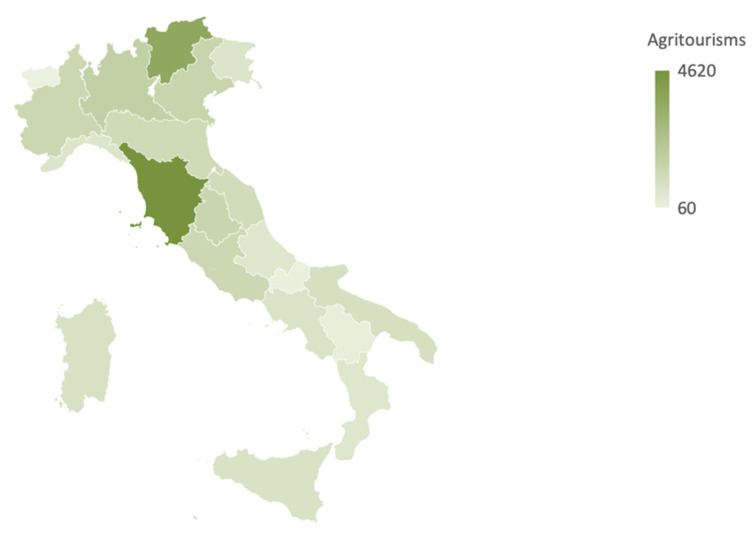
Concentrations of agritourism in Italy. Elaboration based on ISTAT 2019 [2].

The first phase made it possible to identify, in 2018, 23,615 farms with an agritourism vocation on the Italian territory. In the regions of Central Italy there is a greater concentration, especially in Tuscany (4620 units), Umbria (1402 units), Marche (1082 units), Lazio (1278 units), followed by regional surveys in Puglia where there are 876 companies, focusing on Salento (over 60% of the entire region) (Figure 2) [2].

**Figure 3:**
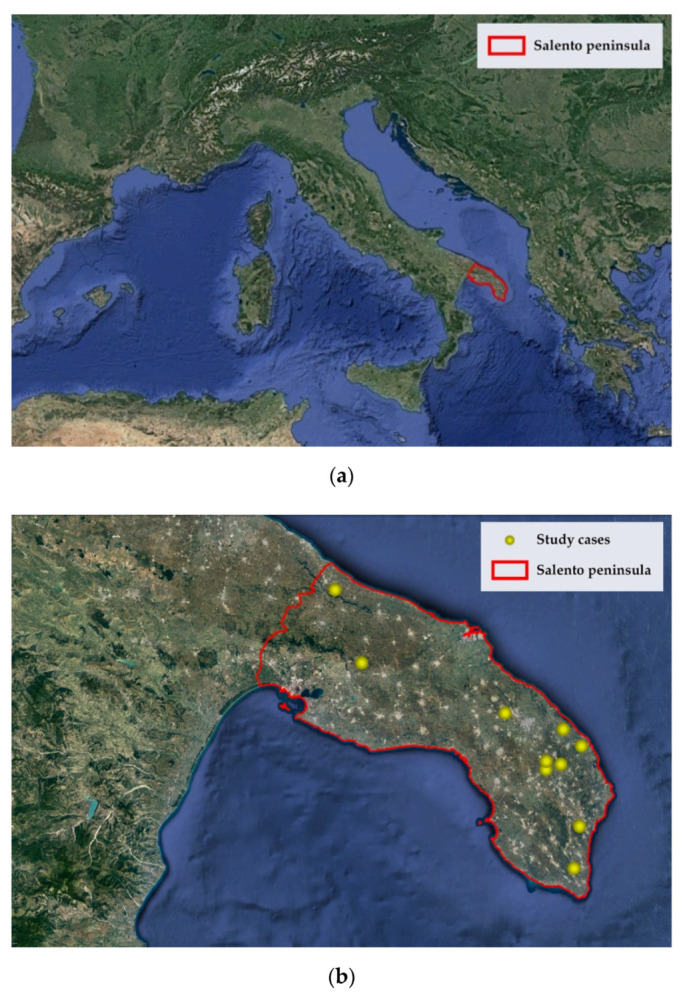
Study area: (**a**) Salento peninsula in the Puglia region, Italy; (**b**) 10 case studies identified.

In order to identify relevant case studies, in fact, the survey focused on agritourism in Salento, a vast territory within the Puglia region, in southern Italy (Figure 3a). The study area was selected for convenience, due to the geographical proximity of researchers, which made it possible to accurately collect information in the field with positive effects on the reliability and validity of the achieved results. Furthermore, thanks to its historical, cultural, and environmental heritage and its idiosyncratic traditions, Salento has a very strong identity which, better than other territories, can help the agritourism develop innovation using a tradition-based strategy.

In addition, for the identification of the 10 case studies (Figure 3b) (see Appendix A for details), the following selection criteria were used: (1) relevance and consistency of the business object with respect to the specific purposes of the study; (2) relevance and completeness of the available information; (3) homogeneity of the temporal phases (all the investigated agricultural enterprises have experienced a start-up phase and a development phase of the agritourism activity, grounded in sustainable innovation through traditionally based strategies); and (4) homogeneity in the degree of awareness and respect for the principles of sustainability, and a propensity to invest in actions aimed at sustainability (national organic certifications).

The information gathered in the first phase of this research was sourced from multiple public sources. This preliminary stage was highly investigative, in terms of collecting information which is directly or indirectly referred to the very core of this study—that is, the way agritourism pushes innovation by way of tradition-based methods. This phase is essentially a thorough study of documented sources and archives.

The second phase of the research concerned the collection, analysis, and interpretation of the data, obtained mainly through direct interviews and the administration of semi-structured questionnaires to agritourism owners/entrepreneurs (Table A1).

Finally, the answers to the interviews and questionnaires were compared with each other with the aim of identifying similarities and differences, and the results of this process were subsequently triangulated [111] with those deriving from other sources, in order to develop robust and consistent causal relationships and strengthen the validity of the results of the case studies [114,115].

Overall, the survey made use of the following sources: interviews (de visu and telephone calls with farmers or family members involved in the activity); e-mail questionnaires (exclusively for owners/entrepreneurs); information documents and institutional databases (ISTAT, CCIAA, Confagricoltura and InfoCamere); websites of the investigated agritourism companies; and the regional regulations in force on the subject.

This array of diverse sources reduced the restrictions deriving from each source if considered separately, as “the most important advantage which occurs using multiple sources of evidence in development of converging lines of inquiry” [116]. Namely, our first-hand sources affirmed and expounded what developed from the auxiliary information and data, in this way expanding our confidence on the outcomes.

#### 3.2.1. Documentary Information

As for the documents that were studied, we had access to multiple sources, for instance specialized travel blogs, several reports and interviews gathered from the local, national, and international press, along with scientific journals. The main contribution deriving from the documentary information is an understanding about the concept and characteristics of agritourism.

#### 3.2.2. Archival Records

With regard to online-available archives, we sourced from wwww.masseriesalento.it, selecting only agritourism with organic/bio certifications, as well as from the websites of the individual selected agritourism entities. The homepages of the selected agritourism entities offered information about the property features, their agritourism history, and their value assessment. This source of archival records provided the authors with the construction of a conceptual map to highlight the common characteristics of agritourism, as well as specifically looking for any relationship between tradition, innovation, and strategy.

#### 3.2.3. Information from the Survey 

**Figure A1:**
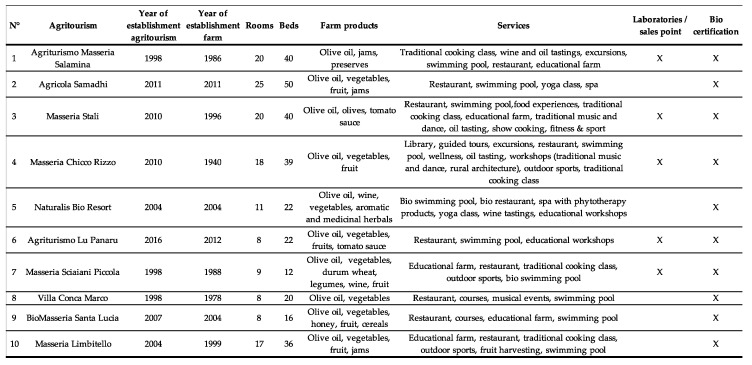
The 10 case studies.

About information from the survey, all 10 agritourism entities in the sample (Figure A1) were contacted by telephone and e-mail to explain the purpose of our research. The concepts of authenticity, tradition, identity, sustainability, and innovation were defined to the interviewees, who were the agritourism owners/managers. All the interviewees filled out an open-question online questionnaire (Table A1). This kind of source served to deepen the understanding of the relationship between tradition and innovation in agritourism. Results were aggregated and compared with the findings of other authors, so as to establish congruence and determine the level of agreement regarding the agritourism concept.

#### 3.2.4. Direct Observation

Finally, with reference to direct observations, three agritourism samples were visited in informal conditions (participating in special events), which led to a better collaboration with the proprietors and the workforce. On several occasions, we also interacted with the guests of these three agritourism samples, which led to a thorough knowledge of the experience as seen from a tourist perspective. In all instances, we took written notice of our findings during or shortly after our stay. The direct observations helped the authors in providing previous results from other data sources and for a general understanding.

### 3.3. Data Analysis

According to Strauss and Corbin (1998) [117], the analysis of data followed an inductive and iterative process. We have studied the 10 cases with reference to the various sectors of innovation where tradition can be an engine of inspiration for the agritourism. We classified diverse sources, so as to attain a thorough comprehension of the cases, organizing the work so that all data was compared. This procedure led to fully identify the different facets associated with traditional resources, and the effective recombination of these traditional resources to generate innovation.

We discuss the interpretation of such information as applied to our context in order to identify possible associations between tradition and innovation, attaining a comprehension on how important traditional resources are to deploy innovation strategies within agritourism.

Following Eisenhardt (1989) [112], a sequence of reiterations were performed with regard to our data, both primary and secondary, reading them under the perspective of innovation literature, so as to better focus on emergent discoveries and thus boost the theoretical bases of analyzed topics.

### 3.4. The Setting

Agritourism establishments are a business model oriented towards sustainability and highly appreciated by tourists: it enhances rural areas, often located outside traditional tourist circuits; it is authentic in its experiential contents (using places, offering services, and involving locals subjects and tourists); it financially supports agriculture and the local economy by stimulating the processes of social growth and economic development; it protects and brings to life past traditions and the ancient peasant culture; and it innovates through tradition.

The physical spaces that host the farmhouse are often ancient farms from the 17th, 18th, and 19th centuries, knowledgeably restored, and respecting the authenticity of the place and the ancient traditions of the peasant culture.

The key requirements of agritourism are (a) the presence of indigenous crops, the production of organic food, and the catering quality; (b) the presence of local craftsmanship that preserves the authenticity of the place; (c) a very strong and inclusive rural community; (d) a management structure overseen by the owner; (d) an “authentic” environment made up of spaces renovated and furnished according to tradition, while guaranteeing every comfort; (e) the lack of a professional management standard, consistent with the proposal of authenticity of experience and with the roots in the community and throughout the region; and (f) a recognizable style, an identity, a common feeling that identifies “that” place, differentiating it from all others.

## 4. Results

The case analysis involved 10 successful agritourism entities located in rural areas of particular cultural and naturalistic interest within the Salento area, in the province of Lecce and Brindisi in Puglia, a region of southern Italy. Consistent with the exploratory aim of the research and with the qualitative methodology described, our research revealed an array of particularly interesting arguments, namely how tradition is able to drive innovation across different agritourism concerns. We categorized our findings according to three classifications: (1) the role of tradition; (2) the source of tradition; and (3) the recombinant strategies. The main outcomes of this grouping allowed to properly address the key demands of our research:

▪ How can agritourism entrepreneurs be inspired to innovate through tradition, in the wake of sustainability?
▪ What are the sources of tradition that lead to an innovation shift, in the wake of sustainability?
▪ How does agritourism contribute to the local development process and to create public value?

### 4.1. The Role of Tangible and Intangible Traditional Resources

Agritourism represents a particular type of genuine and rural hospitality that use traditional and local resources, a culturally precious atmosphere, and conveying a more resident-type approach rather than a tourist-oriented lifestyle [118]. In its processes, agritourism controls and uses traditional tangible or intangible resources: the former are rather all-embracing, as resources are the historical attractive points that lead tourism towards the “physical space” of a community or an entire region. Historic edifices, ancient monuments, works of art, artisanal artefacts, or an old peasant village are fine examples of tangible assets [119]. Intangible assets, on the other hand, express the identity of a specific territory, or refer to a particular activity related to its heritage, its culture, and folklore, capturing the *genius loci* [120].

The owner of Agritourism 6 speaks of the guests: “*This place attracts people with a strong passion for history, for ancient traditions, looking for new experiences. Guests consider authenticity a very real value in today’s world*”. Therefore, the value is given by the coherence with the context and by the stretch of authenticity that reigns in this place, “*while providing them with every comfort, we wanted the rooms obtained from the ancient stables to be decorated in a manner consistent with the local style and context of countryside.*”

Agritourism 5 also has a spa housed in the open countryside, which incorporates an ancient cistern. Here, herbal treatments are performed using products that derive from the crops of the same agritourism. This is how the owner expresses himself: *“The fruits of our garden and the biological cultivation of aromatic plants, with their variety of colors and scents, capture the senses, letting the guest enjoy the flavors of the past both in the food and in the soul*. *For this reason, I also created a “perfume” with the intention of capturing the soul of this land.”*

The usefulness of tradition, in relation to the concept of “nostalgia” for better times, is clarified by sociologists and psychologists: consumers are inclined to seek comfort in less chaotic places and in environments connected to more culturally stable times than those in which they are forced to live today [121,122,123], and search for simplicity [124,125].

The enhancement of agritourism, as of ancient palaces or historic boroughs, is an amalgamation of innovation methods concerning both the product (the edifice that is preserved and renewed to be turned into a place of hospitality) and the offered services (such as experiential itineraries and the shopping experience of typical products) [126].

The owner of Agritourism 4 tells us: *“Our farmhouse, dating back to the 1700s, was formerly used as a post station for horses. Positioned on old sheep tracks and local roads, which from Roca reached Lecce, and from Otranto to Gallipoli. Today we let our guests follow the same itinerary in the countryside, which has remained intact in its identity beauty. The guests tell us that it seems to go back in time.”*

In agritourism, traditional experiences and accommodation services merge, resulting in a recombination process that is an essential part of the local heritage. Agritourism can thus represent a significant, genuine cultural marker, both for its historical and its ethnic legacy [125]. In addition, guests can often experience a close relationship with the local culture, delivering an authentic representation of native communities.

Agritourism 9 organizes traditional cooking courses, events with traditional local music and courses to teach the “pizzica”, a folkloristic dance.

Agritourism 5 organizes meetings with local artisans who safeguard ancient traditions to be preserved and handed down. The owner remarks: *“For our guests, we organize meetings with local artisans, for example with artisans who work the ancient textile art of the bobbin. Knowing the ancient, guests discover something unusually new.”*

Tradition can be built and conveyed in a tangible or intangible manner. Seen as a whole, Agritourism 1 exploits both tangible and intangible resources. For instance, apart from its accommodation facilities, a wide array of recovered furniture pieces is evident (old wagon wheels, ancient sideboards, and ancient equipment used in the countryside, e.g., an old plow).

In principle, the concept of agritourism is based on renovation as an integral part of the place’s history, for instance keeping the original building’s features or preserving the methods and ingredients of ancient local recipes, which means keeping alive the deeper significance of these places.

The owner of Agritourism 6 said: *“In the redevelopment of our buildings we followed the local tradition, respecting the identity of the original architecture and the furnishings, but we did more; our agritourism is part of a story that integrates with the surrounding area. The ancient complex is located along the path of the Via Francigena and was used in the XII century AD as a stopping point for pilgrims who headed to the Holy Land. We tell this story to guests by offering them an intimate experience, both material in living the small peasant village, and emotional in letting them discover its cultural and value heritage.”* The owner continues: *“We lead guests to relive a historical experience in walks along the ancient route, still today as then delimited by dry stone walls.”*

A quote from UNESCO website page confirms this: the ancient dry stone walls of Salento are strongly identifiable and authentic artistic elements, so much so that since November 28, 2018, they have become part of the list of UNESCO Intangible Cultural Heritage because they represent “a harmonious relationship between man and nature”.

### 4.2. The Sources of Tradition

Following the proposal of Presenza et al. (2019) [45], results emerging from the empirical evidence allow to group the tradition sources that inspire innovation into four categories: (a) traditions deriving from the agritourism itself; (b) traditions from a past industry; (c) traditions of a specific period of time; and (d) traditions from the destination.

Concerning a tradition that derives from the agritourism itself, most Italian agritourism entities are small family-run businesses, meaning that one or more families own and/or manage the properties and businesses [126].

The owner of Agritourism 4 recalls that *“The Masseria originally belonged to the noble Granafei family of Sternatìa. In the years 1920–30 my grandfather was a farmer, then my father in 1961 managed to buy the farm and the land surrounding it, planting his business here with great passion and gratification. It was a great personal satisfaction for my father, he put a lot of commitment in his company, with which we sons also collaborate today.”*

So, from the owner’s point of view, agritourism is experienced as a model of enterprise distinguished by personal satisfaction, giving the local community high levels of passion, sacrifice, and commitment for the business.

Tradition can also derive from an old industry sector. Agritourism is able to leverage on ancient buildings that can no longer fulfil its original purpose, and that stayed vacant or neglected for ages. Agritourism 5 has recovered an ancient barn from the 19th century with the aim of creating iconic suites.

Agritourism 9 has recovered an ancient underground mill. The farm is built on a 5th century A.D. settlement previously owned by the Basilian monks, and has been called “Agrimuseum of Puglia”, because over the centuries an ancient noble villa and various rural structures and shelters for households and animals have been superimposed on the ancient underground mill.

Tradition can derive from a particular time period. Agritourism 9 allows an immersion into the 5th century AD. It is located in the territory of the Macurano rock settlement park, an archaeological site of great value, as it preserves the evidence of settlement forms typical of the Greek–Byzantine culture, subsequently transformed into underground oil mills. There is a typical ancient local building called “pajara” built exclusively using dry stones, in which a cistern attached to the remains of a tank where the grapes were pressed is visible. The hypogeum oil mill is located in an ancient cave where the remains and wheel of an ancient olive mill are visible.

In some cases, agritourism has been established by restoring important historic edifices or ancient peasant villages. This is the case of Agritourism 5, where the exclusive charming residence was built from the wise renovation of an ancient 18th century peasant village surrounded by typical dry-stone walls.

Finally, sometimes the destination is key to successfully positioning an agritourism endeavor. For instance, a farm can push its image and its value on the market by merging tangible (e.g., culture, architectural heritage, agriculture, and crafts) and intangible (e.g., wellbeing and traditional lifestyle, social bonds, etc.) traditions, seen as destination-derived assets able to convey a genuinely authentic message.

Agritourism 4 is a typical case of a “destination” serving as the source of tradition. As it happens here, the confines of tangible and intangible tradition are rather difficult to draw.

The agritourism is located in the Sternatìa countryside, in the middle of a territory called “Grecìa Salentina”, a Hellenophone linguistic island of Salento in which a dialect known as “Griko” is spoken. The Greek penetration in the Salento peninsula took place both in ancient times (Magna Graecia) and with the subsequent Byzantine domination, in particular with the emigration of religious clerics during the disputes over the iconoclasm in the VIII century AD. In 1990, a cooperation process took place between the Hellenophone-speaking municipalities to enhance and promote the Grika culture and traditions, then through a law, the Consortium of the Municipalities of the Grecìa Salentina was officially established while the Association of the Municipalities of the Grecìa Salentina continued the aim of promoting knowledge of the Greek area of Salento, safeguarding the culture, the language, and the identity of this territory.

Furthermore, this area is very attractive for tourists because it is immersed in an open-air archaeological park, with important sites of dolmen and menhir, megalithic monuments, made up of stone blocks that perform the function of collective tombs.

The whole territory draws on ancient culinary traditions and many typical food outlets have mushroomed. For example, in the countryside of Zollino, one of the countries of the “Grecìa Salentina”, the typical dwarf pea is grown, a unique local legume that is eaten dry, gaining the recognition of the Slow Food Presidium and now included in the list of Apulian Traditional Agri-Food Products.

Agritourism 4 draws on typical local resources. Its restaurant sources all varieties of produce from local farmers, offering a genuine gastronomy, rich in traditional flavors, enriching the destination identity and preserving both history and the *genius loci*.

Even the owner of Agritourism 7 confirms a careful connection to the destination, affirming: *“the restoration of the agritourism and the reception were inspired by the recognition of a historical heritage with marked identification characteristics. They have been revitalized by adopting a rigorous approach to conservation.”*

### 4.3. The Recombinant Strategies

Recombining together more than one tradition is a creative and competitive method of using it to strategically innovate processes [64,127]. The goal is indeed to incorporate its diverse components so as to generate an articulate entirety [128].

The analysis of cases on how traditional resources merge towards innovation points to the success of each individual agritourism, achieved through the search for different interdependent components that often share geographical proximity [38].

Therefore, an agritourism should be able to reshape segmented (or sometimes entire) value chains to build the new product of the experience to offer to guests. This means taking into consideration the origins of historical assets, as well as boosting activities and trends that make the tourist experience an authentic one, since the ultimate purpose is to connect and integrate the diverse facets that create a coherent whole [128].

The value of the interdependencies [76] has clearly emerged in the case of agritourism, offering a chance (and at times the necessity) to arrange traditional resources in a new way. In fact, the evidence that emerges from the research is that agritourism is not merely concentrated on refurbishing existing buildings as an end in itself, but is rather interested in a robust and long-lasting amalgamation with the surrounding community and territory. This underlines the concept of agritourism as a phenomenon that creates the new from the old and involves the guest in more aspects of the daily life of the surrounding premises, proposing a comprehensive array of experiences across a co-evolutionary spectrum [68].

An example of this derives from Agritourism 2: *“our project does not focus only on the restoration of the architectural heritage but also contributes to the increase in commercial activities and services in the area.”*

As has been observed in the literature [68], agritourism is connected to its territory by way of diverse methods of cooperation. Therefore, the multifaceted and dialectical correlation with stakeholders from the local environment becomes fundamental.

More specifically, Agritourism 5 shows a significant involvement in a number of events promoting territorial craftsmanship (textile embroidery of the “tombolo”, the art of the “panari”, hand-woven baskets composed of rushes of olive and cane, traditionally used for the olive harvest, and today a typical souvenir for tourists); enhancement in the catering of local products, such as fruit, vegetables, and medicinal herbs; and everything that can be used to safeguard and showcase local traditions.

The owner of Agritourism 5 remarks: *“With local associations, we organize nature walks and cultural itineraries to let tourists experience the natural and cultural environment. Guests recognize the value of offering something new that starts from tradition.”*

Agritourism 9 explains: *“Through tradition, we pursue the goal of innovating in a sustainable way. For fifteen years we have been a certified organic company, we have composted and meticulously recycled packaging. We only use eco-sustainable soaps, we have solar panels for clean energy, we have restored the farmhouse with natural materials used in antiquity, such as natural lime, water-based paints, stone and wood.”*

Innovation strategies, geared towards the strategic positioning of agritourism, undoubtedly outrun the single company and its owners. As a matter of fact, synergies between businesses and surrounding communities are a distinctive feature often involving a destination in its entirety, thereby delivering a distinctive blend of identity and local entrepreneurship [9].

The owner of agritourism 5, a great connoisseur of ancient crops and medicinal herbs used in cosmetics and phytotherapy, produces the raw materials he uses in the creation of medicinal products in his factory, founded in 1989. The entrepreneur, with his family, is very committed to the defense and enhancement of the area, and creates many initiatives to stimulate awareness on the importance of sustainability and the respect for man and nature. One of the last important initiatives is “Martano city of Aloe”, a true example of collaboration between private and public through the donation and planting of over 3000 aloe plants within all the flower beds and parks of the city of Martano, transforming Martano into the first aloe-city in the world. With these initiatives, he has captured the attention of international guests such as actress Helen Mirren and director Taylor Hackford, international movie stars particularly sensitive to the issue of environmental sustainability. This case, more than any other, proves how agritourism is a model for developing a community project capable of generating effective results when it comes to reputation and image, thus creating development, employment, sustainability, and therefore generating public value.

## 5. Discussion

A review of the literature on innovation exposes how the use of traditional resources and the capacity for innovation can bring significant advantages in different sectors [62,77,129], recently also in the tourism sector [45]. At the same time, a review on agritourism literature, as a phenomenon derived from the multifunctionality in agriculture [14,20], shows no acknowledgment as to the potential for agriculture and tourism that may come from it. Therefore, strategies based on tradition appear to be strongly underrated as to the capacity to boost innovation and the creation of value.

Given the importance that guests increasingly attach to authenticity and experience-oriented travel packages—which are based on significant exchanges with the surrounding community [130]—this gap is particularly obvious and serious. In fact, the combinatory capabilities of every kind of firm are influenced by their internal organizations, as well as by their connections with other organizations, such as providers, customers, and networks of collaborating organizations [76,131,132]. It is known how organizational knowledge is defined by the practices used to structure work and by the interactions between workers [133,134].

The rising attention of travelers towards agritourism can yield a strategic advantage to the tourism and agriculture industry, in its overall vision of multifunctionality and in attracting the interest of scholars on the phenomenon, as a very relevant and promising research field.

This paper focuses on the agritourism sector and its entrepreneurship aspects, with particular emphasis on the way its players are inspired by traditional resources to push innovation across diverse facets of their business. 

Upon analyzing agritourism, we focused on the usage of tradition as having a wide influence on innovation, with reference to products, services, processes, management, and marketing. For instance, native crops are cultivated after thorough research, thus becoming a novel form of sustainable innovation in agriculture; the promotion of local enogastronomic bio-products constitutes a pioneering marketing method; bio-oriented agritourism add to the community value chain by sourcing both materials and services at a local level, thus innovating through sustainable processes.

Our outcomes highlight four main sources of tradition: the firm itself, the knowledge associated with previous industrial activities, a definite period of time, and the destination in which the firm operates.

When the basis is the firm itself, accomplishments originate from its ability to transpose traditional knowledge and activities into novel consumable products/experiences [76]. Hence, success relies on the manager’s/owner’s ability to re-interpret traditional products and processes regarding the enterprise. A second source of tradition is detectable from a specific period of time [79]. In this latter case, the success of a firm depends on its capacity to connect with, and extract value from, a particular historical event, thus defining the significance of a specific timeframe for the destination.

Enterprises may also seek innovation as based on recent or legacy knowledge related to an industrial activity [135]. Utilizing past industrial heritage involves the discovery, regeneration, and repositioning of traditional industrial activities and their related know-how, in order to generate valuable assets arranged and deployed by way of new products or experiences.

Ultimately, the tourism destination itself may be considered as the foundation for inspiration, as a result of its *genius loci* [120], capable of delivering unique and attractive features that foster the diverse local tourism firms, clearly distinguished from other destinations [126].

Our study of ten cases clarifies how entrepreneurs generate value for customers through the revitalization, preservation, and strategic repositioning of tangible assets sourced from a specific tradition. At the same time, intangible assets pertaining to tradition may also significantly influence the market appeal, ultimately achieving success when it comes to agritourism enterprises. Therefore, policymakers and destination managers should be aware of the opportunities to affect both the image and the positioning of the destination, and ultimately the social and economic well-being of the surrounding community by way of attentive policies, with particular regard to the architectural, artistic, and environmental heritage, principally where these are unique and prevalent.

As maintained by Fleming (2001) [136] and Laursen (2012) [137], by investigating the way firms deploy innovation strategies, it appears that the development of novel products is a function of how organizations seek a particular know-how across a wide array of dimensions, experimenting with various recombinations of the attained knowledge. Numerous scholars prove that the novelties derive from the synthesis of existing and widespread technologies [82,128,138,139]. Therefore, inventions can often be described as a combination of previous and/or new technologies [136,140]. This theoretical framework allows us to think of innovation as a recombinant research process for better combinations and configurations of the existing ones (tradition). This is also confirmed by our findings.

**Figure 4:**
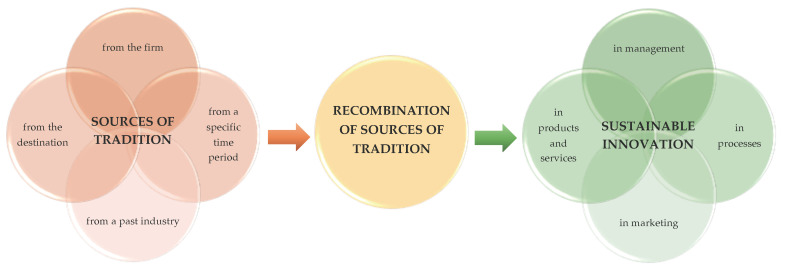
Conceptual model: The process of sustainable innovation through a tradition-based strategy.

Figure 4 is a conceptual model, indicating how sustainable innovation can be sourced from tradition. Following Presenza et al. (2019) [45], the four identified sources of tradition are the firm itself; the historical past of an industry; a specific time period; and the tourism destination.

In the quest for sources of tradition, an enterprise should identify and accumulate resources of value and home in on them. Consequently, the enterprise ought to develop empowering capabilities in order to combine and exploit those resources. Such a recombination course produces new products or even completely new business models, so the firm can adapt and distinguish itself within its market environment. In order to succeed in this feat, the firm must be able to process, interpret, encode, manipulate, and access information in a resolute and goal-directed fashion.

Our findings reveal that the agritourism owners/managers accrue consistent value by recombining traditional resources with other components and assets, associated with their geographic origins (in the destination), hence fostering the co-evolutionary view with regard to the relationship between firms and their environment [68].

Accordingly, in the co-evolutionary perspective, agricultural enterprises and territories are closely interconnected and are conditioned by evolution, since they are functional to others and the other way around [37,141]. In this perspective, two aspects take on particular importance: the territory (meso level) becomes a key agent for connecting agricultural enterprises (micro level) with the rest of society (macro level), extending the interdependencies and positive externalities [37,141,142]; and the systemic setup of both companies and territories is taken as a basic condition to consolidate or renew resources and skills [143], and therefore to adapt to each other effectively, according to a shared strategic orientation.

## 6. Conclusions

This study investigates the topic of innovation strategies based on tradition, in the wake of sustainability, in the agritourism sector as derived from the multifunctionality of agriculture.

The results reveal that the drive to positive outcomes is made successful by the mix of tangible and intangible resources that derive from tradition, used as engines for innovation, as they could also be applied to different tourism sectors and throughout diverse destinations.

Furthermore, the results give a prominent role to tradition deriving from different sources, as it is able to help creating new products and services based on the recombination of tangible and intangible heritage assets, particularly identifying a place, bringing out its authenticity and capturing its soul, making that site even more attractive.

While noticeably setting a logic in the way traditional resources are used strategically, this method also maximizes its results based on new technologies. On the contrary, “the intelligent support of the ‘old’ by the ‘new’ can be seen as a further form of recombination that offers the opportunity to differentiate and strategically reposition—opening more doors to the future” [45].

With regard to the theoretical implications, we believe that this study contributes to literature in filling the absence of a reflection on strategies for innovation, based on tradition, in the specific sector of the agritourism. We have seen how a company has the task to elaborate, interpret, codify, access, and deploy information in an intentional and direct way to the objectives, using authenticity and tradition to generate innovation and sustainability. We believe that a broader application of this concept will generate new and innovative tourism opportunities, with the capacity to attain substantial social and economic benefits for businesses, destinations, and territorial communities, contributing to the creation of public value in a coevolutionary perspective [68].

More specifically, results yield to the rediscovery of traditional assets and their ensuing recombination with complementary resources [80], thus leading to a differentiation of distinguished competencies and thereby generating a competitive advantage in the wake of sustainability [144].

In addition, agritourism could play the role of facilitator, namely in the definition and evolution of a territory, thus bringing back public value.

This study provides a thorough comprehension on how traditional innovation plays a leading role in the development and strategic positioning of tourist destinations. In this regard, innovation based on tradition can also expand the way to achieve sustainable development throughout the territories in which agritourism becomes a meta-organizer [145] or serves as a “knowledge hotspot” [146], because of its ability to encourage local learning processes at a local level and spreading heritage-based innovative processes across other territorial organizations.

With regard to the practical implications, the research offers several opportunities for the development of new experiential tourism businesses that are related to the conservation, protection, and recovery of the tangible and intangible assets related to it. It also represents a vast opportunity to develop particular destinations in a sustainable fashion, especially when the surrounding territory benefits from a global standpoint when it comes to innovation.

From a management point of view, it highlights the elements that can help with strategic positioning and positively influence the creation of new business models [1]. This opportunity is particularly valuable given the growing demand for authentic experiences and historical value in the wake of environmental sustainability.

Of likewise importance is the position that heritage-based innovation attains in revitalizing its surrounding community. From a political point of view, agritourism can grow to become effective promoters for the development of communities and regional areas that place themselves in a different tier if compared to mainstream tourism, and, as such, play an essential role in favor of previously low-appealing areas, with consequent greater inclusion and forms of sustainable tourism in the three environmental, economic, and social dimensions. For this reason, at a practical level this research also provides indications that can be valuable for policymakers.

## 7. Limitations and Future Research Directions

We believe that the limits of this research, on which we will be working, may also serve as opportunities for future studies. First of all, the study has been narrowed to 10 cases in Salento, a vast territory of the Puglia Region, in southern Italy, the country in which the phenomenon of agritourism was initially developed. Whilst being grounded on a clear-cut logic, for the reasons widely expressed in the methodological section, similar studies in other Italian territorial contexts and even more in countries having diverse cultures and national environments can yield different results. In this regard, it would be useful for further studies to verify the generalizability of the results, in the various geographical and organizational contexts.

Secondly, this research starts casting light on the recombinant strategies that agritourism owners have deployed in using tradition as a foundation for innovation, in the wake of sustainability. Further research is needed to better comprehend which factors feed and/or affect recombinant strategies, as is the case of an entrepreneur’s business traits, as well as a series of potential exterior influences, including territorial development policies and the intervention of policymakers.

Finally, the study has been restricted to one type of organization, namely the agritourism business. While it has been argued that the choice of the agritourism also derives from the evidence of a gap in the literature in this context, future research could examine other specific examples in the sector of both multifunctional agriculture and experiential tourism.

## Appendix A

**Table A1:** Questionnaire filled in by the owners/managers of the agritourism entities.

▪ Is the strategy or positioning of your Agritourism linked to tradition, and in particular to any historical characteristic? ▪ Which physical part of your Agritourism is mostly referred to historical characteristics? ▪ Which activities of your Agritourism are mostly referred to traditional characteristics? ▪ What activities of your Agritourism are mostly referred to authenticity? ▪ In the management of your Agritourism, what activities are carried out with respect of sustainability and how do you think tradition can help sustainability? ▪ Is your Agritourism centered on a distinct and substantial identity trait? If so, please provide a description of it. ▪ Describe the relationship between your Agritourism and the territory. ▪ Describe the relationship between your Agritourism and your family tradition. ▪ How significant is it for your Agritourism to safeguard and promote your destination’s history and traditions through a co-operation with territorial stakeholders?
